# Optimized preparation pipeline for emergency phage therapy against *Pseudomonas aeruginosa* at Yale University

**DOI:** 10.1038/s41598-024-52192-3

**Published:** 2024-02-01

**Authors:** Silvia Würstle, Alina Lee, Kaitlyn E. Kortright, Franziska Winzig, William An, Gail L. Stanley, Govindarajan Rajagopalan, Zach Harris, Ying Sun, Buqu Hu, Michael Blazanin, Maryam Hajfathalian, Paul L. Bollyky, Paul E. Turner, Jonathan L. Koff, Benjamin K. Chan

**Affiliations:** 1 Yale Center for Phage Biology and Therapy, Yale University, 165 Prospect Street, New Haven, CT 06520 USA; 2 Department of Ecology and Evolutionary Biology, Yale University, New Haven, CT 06520 USA; 3 Department of Internal Medicine, Section of Pulmonary, Critical Care, and Sleep Medicine, Yale School of Medicine, New Haven, CT 06519 USA; 4 Technical University of Munich, 81675 Munich, Germany; 5 Division of Infectious Diseases and Geographic Medicine, Department of Medicine, Stanford University, Stanford, CA 94305 USA; 6 Program in Microbiology, Yale School of Medicine, New Haven, CT 06520 USA

**Keywords:** Bacterial infection, Coevolution, Bacteriophages

## Abstract

Bacteriophage therapy is one potential strategy to treat antimicrobial resistant or persistent bacterial infections, and the year 2021 marked the centennial of Felix d’Hérelle’s first publication on the clinical applications of phages. At the Center for Phage Biology & Therapy at Yale University, a preparatory modular approach has been established to offer safe and potent phages for single-patient investigational new drug applications while recognizing the time constraints imposed by infection(s). This study provides a practical walkthrough of the pipeline with an *Autographiviridae* phage targeting *Pseudomonas aeruginosa* (phage *vB_PaeA_SB,* abbreviated to ΦSB). Notably, a thorough phage characterization and the evolutionary selection pressure exerted on bacteria by phages, analogous to antibiotics, are incorporated into the pipeline.

## Introduction

While antibiotics have contributed to significant increased life expectancy in the last century^1,2^, the misuse and overuse of antibiotics jeopardize their effectiveness^3,4^. The emergence of antibiotic resistance and cross-resistance due to the evolutionary pressure by antibiotics is one of the major threats to human health, particularly to those with compromised immune systems^3,5^. The six leading pathogens that cause mortality associated with antimicrobial resistance are *Escherichia coli, Staphylococcus aureus, Klebsiella pneumoniae, Streptococcus pneumoniae, Acinetobacter baumannii,* and *Pseudomonas aeruginosa*^6^, and in 2019 these pathogens were responsible for over 250,000 worldwide deaths associated with antimicrobial resistance^6^.

Felix d’Hérelle first published the clinical use of bacteriophage (phage) therapy in 1921^3,7–9^, and since then compassionate phage therapy has been applied to treat bacterial infections when standard, approved therapies fail^10–14^. The emerging antibiotic resistance pandemic has renewed attention in phage therapy, which uses lytic phages (viruses) that specifically infect bacteria and force them to switch their metabolism from growth to phage production^3^.

Phages are abundant in nature and within microbiomes, outnumbering bacteria worldwide by an estimated factor of 10^2,15^, which suggests the ability to find phages for antibiotic resistant bacteria. In addition to this host-specificity, phages are self-amplifying, and have the ability to disrupt biofilms, which are important characteristics to target resistant, persistent bacterial infections^3,16–20^. However, despite numerous efforts in recent decades, the introduction of phages as approved drugs has been challenging due to phage diversity, narrow spectrum of activity, and challenges to produce a long-term, stable biologic^21–26^. In the U.S. phage therapy occurs via a single-patient investigational new drug (SPIND) application to the Food and Drug Administration (FDA) under the FDA’s expanded access IND (eaIND) program^27,28^. FDA eaIND review includes the clinical indication(s), protocol for phage therapy, phage manufacturing, and the proposed consent form. Individuals cannot be candidates for existing clinical trials, which is relevant as more clinical trials are available for phage therapy. Institutional Review Board (IRB) approval or an IRB waiver is required.

This study focuses on the steps to develop phages for SPIND eaINDs using a phage targeting *Pseudomonas aeruginosa. Pseudomonas aeruginosa* is an opportunistic pathogen that is notoriously difficult to manage because it is frequently resistant to antibiotics^29^. While *Pseudomonas aeruginosa* is found in soil and water, it is an increasingly common human pathogen in immunocompromised patients, hospital-acquired or medical device infections, and in lung disease with impaired mucociliary clearance^30,31^. *Pseudomonas aeruginosa* requires antibiotics for treatment, and unfortunately the effectiveness of our current antimicrobial arsenal is dwindling due the ability of *Pseudomonas aeruginosa* to form biofilm and other mechanisms that increase the prevalence of antimicrobial resistance^6^. Antibiotics alone are increasingly insufficient in some patient cases, and adjuvant therapeutic strategies, such as phage therapy, are imperative for patients who do not respond to conventional medical approaches. Beyond phage therapy, other alternative methods for addressing antibiotic-resistant or recalcitrant *Pseudomonas aeruginosa* are being explored, such as vaccines, antimicrobial peptides, immunotherapy, iron chelators, and quorum sensing inhibitors^31,32^. However, none of these have been approved for clinical use to date.

**Figure 1:**
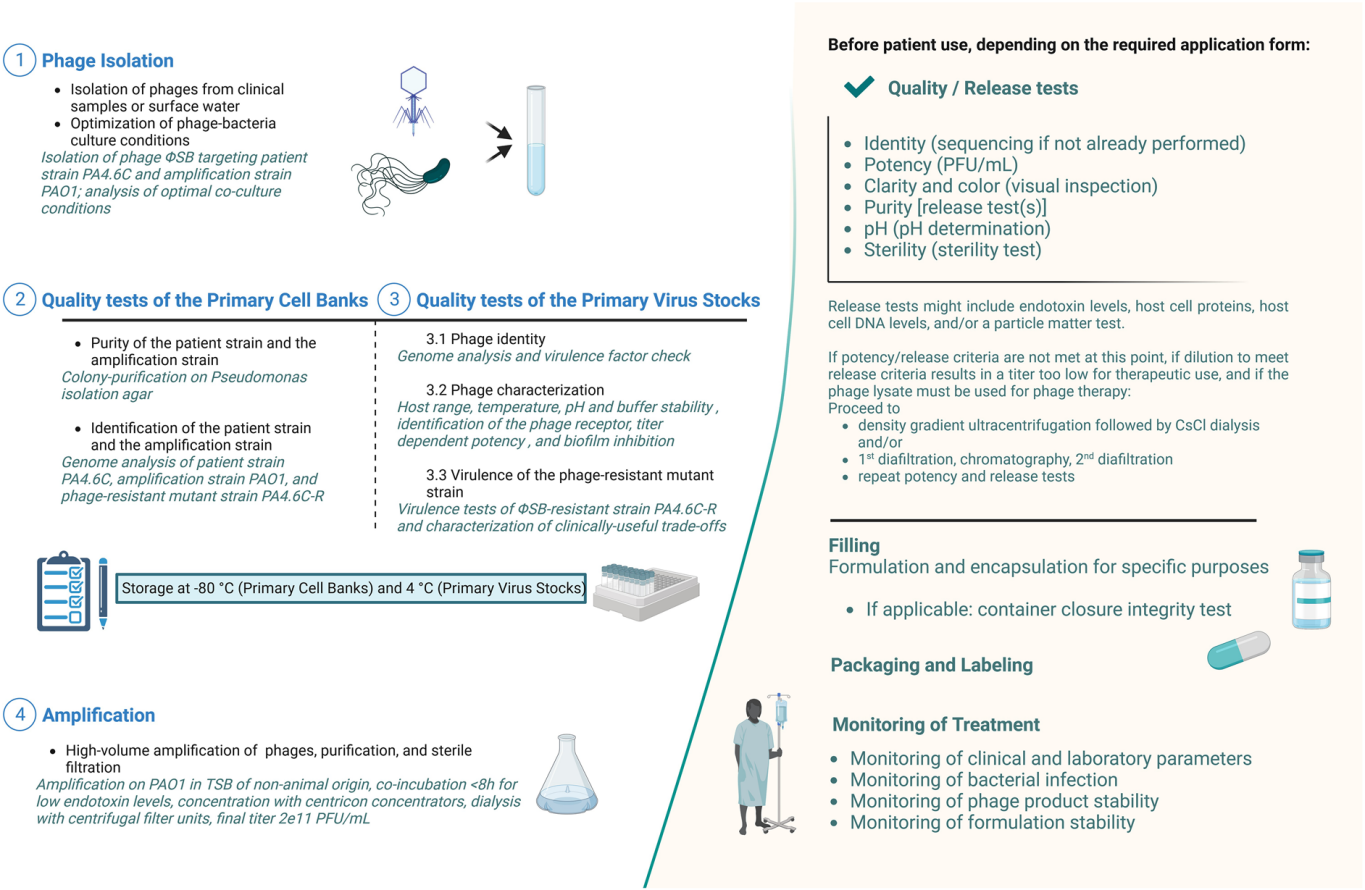
Pipeline of phage preparation. *Italic* descriptions indicate the preparation readouts of the optimal phage candidate of this study. CFU, Colony Forming Unit; CsCl, Cesium chloride; PA4.6C, clinical *Pseudomonas aeruginosa* strain to be targeted with phage therapy; PFU, Plaque Forming Unit; TSB, Tryptic Soy Broth; ΦSB, phage *vB_PaeA_SB*. Created with Biorender.com.

At Yale University’s Center for Phage Biology & Therapy, a preparatory modular approach has been established to produce safe and high-quality phages for SPIND eaINDs (Fig. 1), while recognizing that patient clinical condition may also affect time constraints. This pipeline starts from phage isolation (step 1) and includes a thoroughly characterized Primary Cell Bank (PCB, step 2), Primary Virus Stock (PVS, step 3), and production of a high-titer phage solution (step 4). Notably, we propose to include the evaluation of useful evolved trade-offs in phage-resistant bacteria because phage therapy exerts selection pressure for target bacteria to evolve phage resistance^33^. This analysis also excludes deleterious trade-ups, potentially caused by phage selection pressure^22^, as an integral part of the pipeline, which should be included in modern approaches to developing phage therapy^34,35^.

## Materials and methods

### Bacterial culture conditions

The laboratory strain *Pseudomonas aeruginosa* PAO1 and the PAO1 transposon mutant library were kindly provided by B. Kazmierczak (Yale University) and C. Manoil (University of Washington, Seattle), respectively. The mutants were previously created as detailed by Jacobs et al.^36^. Mutants of this library were selected for this study based on 26 candidate surface phage receptors. De-identified clinical bacterial strains were collected from clinical microbiology laboratory after informed consent was obtained. All strains of this study are listed in Supplementary Table S1. All laboratory experiments were performed in accordance with Yale University’s approved laboratory protocols. For subjects that proceed to bacteriophage therapy, FDA eaIND and Yale University Human Investigation Committee/Institutional Review Board approval is obtained.

All bacteria for this study were cultured with shaking at 37 °C in Luria Bertani broth (LB; 1.5% w/v bottom agar, 0.75% w/v soft agar). The Primary Cell Banks (PCB) of bacteria were aliquoted as homogenous collections and stored at − 80 °C in glycerol. Working PCB stocks were obtained from culturing one vial of the homogeneous PCB at 37 °C in LB overnight. For phage host range testing 52 *Pseudomonas aeruginosa* and 6 *E. coli* were included in this study.

### Master virus stocks

Phage *vB_PaeA_SB* (hereafter abbreviated: ΦSB) was isolated from local wastewater (New Haven, CT, USA), amplified on PAO1, plaque-purified three times and re-amplified on PAO1 with a multiplicity of infection (MOI) of ~ 0.01 in Tryptic Soy broth of non-animal origin (Merck, Rahway, NJ, USA) by co-incubation for 6–8 h with shaking at 37 °C, followed by centrifugation, sterile filtration (0.22 µm), and centrifugation with Amicon Ultra-15 Centrifugal Filter Units (100 kDa, Millipore Sigma, St. Louis, MO, USA). Purification by CsCl step density gradient was performed as previously described^37^ at 38,000 rpm for 3 h with four dialysis steps using Amicon Ultra-15 Centrifugal Filter Units (100 kDa, Millipore Sigma, St. Louis, MO, USA) at 4000×*g* for 15 min^38^ and subsequent sterile filtration. The virus family for each phage was predicted using BLASTn search^39^ and confirmed by transmission electron microscopy. Primary Virus Stocks (PVS) aliquoted as a homogenous collection were stored at 4 °C in PBS. For phage amplification (Step 4; Fig. 1), a vial of PVS was amplified on PAO1 (MOI of ~ 0.01) in Tryptic Soy broth of non-animal origin as described above, using Centricon plus-70 concentrators (100 kDa, Millipore/Sigma, Burlington, MA, USA). After completing isolation, characterization, amplification, and purification (Steps 1–4; Fig. 1), the solution was ready for preparation for phage therapy, which is beyond the scope of this manuscript, and includes: dilution to the appropriate titer and buffer, release tests, and formulation for the particular application (e.g., inhaled, intravenous, or topical administration). Endotoxin testing and sterility are confirmed via external testing according to U.S. Pharmacopeia (USP < 71 >)^40^, and while these methodologies are not included, they are discussed below because they are an integral part of the pipeline for phage therapy SPIND eaINDs.

### Quality tests for primary cell banks (PCB) and primary virus stocks (PVS)

Identity and purity of PCB was performed by colony-purification on *Pseudomonas* isolation agar (Neogen, Lansing, MI, USA) and sequencing. PCB potency was confirmed by CFU/mL. PVS was confirmed by genetic sequencing and phage characterization. Phage amplification was performed on PAO1, and titer was measured by PFU/mL.

### Electron microscopy

The size and morphology of phages were examined with transmission electron microscopy (TEM) using a JEOL JEM1400 (JEOL USA Inc., Peabody, MA, USA) at 80 kV. Briefly, 5µL of diluted phage solution were placed onto carbon-coated copper grids (FCF-200-Cu, Electron Microscopy Sciences, Hatfield, PA, USA). After 3 min, the grid was dipped into a ddH_2_O droplet and then 1% uranyl acetate for staining was added to the sample and allowed to dry for 15 min before performing microscopy.

### Phage stability assays

Temperature, pH (buffer adjusted with NaOH and HCl to pH 1–14), serum/plasma, saline, and long-term stability assays were performed with PAO1 at 4 °C except forthe thermal stability assays. Serum was obtained from a healthy donor and single-donor plasma was obtained from Innovative Research (Novi, MI, USA).

### Bacterial growth kinetics

Fresh stationary phase cultures were diluted to an optical density at wavelength 600 nm (OD_600_) equal to 0.1 in LB and cultured in an automated spectrophotometer (BioTek Epoch2 microplate reader, Agilent, Santa Clara, CA, USA) for 20 h with shaking at 37 °C. Measurements at OD_600_ were obtained every 5 min. Growth curve data were fit to a logarithmic curve using the R package GrowthCurver, and growth parameters were averaged over all replicates^41^.

### One step growth curve and adsorption assay

Three vials of 4.5 mL fresh PAO1 cultures and 0.5 mL phage were cultured with shaking (250 rpm, 37 °C) at indicated MOIs (PFU/mL)/(CFU/mL). At each timepoint, 100 µL of the phage-bacteria suspensions were diluted 1:10 eight times and all dilutions were spotted on PAO1 within soft agar. Adsorption assays followed the same procedure as one step growth curves aside from centrifugation of 100 µL of the phage-bacteria suspension at each timepoint at 12,000rcf (relative centrifugal force) for 5 min and diluting the supernatant to assess the free phages in the suspension.

### Biofilm production and eradication

Biofilm assays were performed to estimate: (1) biofilm formation in the presence of phages, (2) the capability of phages to disrupt pre-formed biofilm, and (3) the virulence of ancestral and post-phage evolved bacterial strains. Biofilm assays were adapted from B. M. Coffey and G. G. Anderson^42^: 1.5 mL of fresh bacterial culture diluted to ~ 1e7 CFU/mL overnight was added to 24-well plate (n = 6) and incubated under static conditions at 37 °C for 24 h^43^. To assess biofilm formation (#1 above) phages were added to a final concentration of 1e9 PFU/mL in each well. To assess biofilm disruption (#2 above), plates were washed after 24 h to remove planktonic cells, and phages were added to a final concentration of 1e9 PFU/mL in each well. To investigate virulence (#3 above), the ancestral strain (PA4.6C) was compared to the evolved phage-resistant strain (PA4.6C-R) without the addition of phages. After 24 h, cultures were washed and stained with 125 µL 0.1% crystal violet stain per well, followed by 150 µL 30% glacial acetic acid addition and measured at OD_550_. CFUs were measured on separate plates by gently scratching each well after washing steps and counting on *Pseudomonas* isolation agar (Neogen, Lansing, MI, USA).

### Evolution of a phage-resistant *Pseudomonas aeruginosa* strain

The phage-resistant *Pseudomonas aeruginosa* strain (PA4.6C-R) was evolved by liquid co-cultivation of PA4.6C with phage ΦSB overnight until the culture was not visibly clear, which equaled co-cultivation twice overnight^34^. PA4.6C-R was subsequently colony-purified in triplicate, and resistance to phage was confirmed by plaque assay and co-incubation growth curves in triplicate.

### Antibiotic sensitivity testing

Antibiotic sensitivity to cefiderocol (MedChemExpress, Monmouth Junction, NJ, USA) and minimum inhibitory concentrations (MIC) for piperacillin/tazobactam, ciprofloxacin, cefepime, meropenem, and imipenem (Liofilchem, Waltham, MA, USA) were obtained by recording the lowest concentration of antibiotic that inhibited growth of bacteria on Mueller Hinton agar (Neogen, Lansing, MI, USA), as described by the manufacturer’s protocol.

### Bacterial stimulation of THP-1 cells to produce IL-8 and LDH

PA4.6C and PA4.6C-R cell culture supernatants were obtained by centrifugation of fresh overnight cultures at 4000×*g* for 15 min. The pellet was washed twice with PBS, normalized to OD_600_ = 0.5, resuspended in LB, and centrifuged at 4000×*g* for 15 min. The supernatants were frozen at − 80 °C.

Human monocytic THP-1 cells (American Type Culture Collection, Manassas, VA, USA) were maintained in RPMI 1640 medium supplemented with 10% heat-inactivated fetal bovine serum (Gemini Bio-Products, West Sacramento, CA, USA), 2 mM l-glutamine, and 100 U/mL penicillin/streptomycin (Sigma Aldrich, St. Louis, MO, USA) at 37 °C in a humidified atmosphere of 5% CO_2_. THP-1 cells were differentiated to macrophages by stimulation with 5 ng/mL phorbol-12-myristate-13 acetate (PMA, Sigma Aldrich, St. Louis, MO, USA) for 48 h in 24-well plates at a density of 1e6 cells/well in a volume of 1 mL/well^44^. Microscopy confirmed cell attachment to each well after washing.

THP-1 s were washed twice with PBS and treated with negative (RPMI, PBS, LB) and positive (lipopolysaccharide) controls, as well as the PA4.6C and PA4.6C-R supernatants, and the ΦSB at 1e11 PFU/mL, all in a 1:10 dilution in triplicate. After incubation for 24 h, cell culture supernatants were collected and stored at − 80 °C. IL-8 was measured by ELISA (R&D Systems, Minneapolis, MN). Cell cytotoxicity was measured using a lactate dehydrogenase (LDH) kit (Roche, Basel, Switzerland) as described previously^45^.

### Quantification of the bacterial products pyocyanin and elastase

The blue-colored phenazine pyocyanin was measured by spectrophotometry^46^. Overnight cultures of test bacteria were standardized to OD_600_ = 1.0 before inoculating in 6 mL of LB with 1:100 dilution in 15 mL tubes. After 48 h, cultures were normalized (OD_600_) reflecting about 1e8 CFU/mL and supernatants were collected by centrifugation at 4000 rpm for 15 min. Subsequently, 2.5 mL of chloroform (AmericanBio, Canton, MA, USA) was added to 5 mL supernatants, vortexed 5 × 5 s, and centrifuged for 15 min at 4000 rpm. The bottom (blue) layer was mixed with 500 µL of 0.2 M HCl, vortexed 5 × 5 s and centrifuged for 5 min at 4000 rpm. 100 µL of the upper (pink) layer were transferred to a 96 well plate for spectrophotometric measurements at OD_520_. Pyocyanin concentration (µL/mL) was calculated by subtracting the blank value (0.2 M HCl) from the sample values then multiplying the values by 17.072^47,48^.

*Pseudomonas aeruginosa* elastase was measured with a fluorometric assay as indicated by the manufacturer’s protocol (EnzChek Elastase Assay Kit, Thermo Scientific, Waltham, MA, USA).

### Motility assays

Twitching motility was assessed for PAO1 using a type IV pilus knockout strain of the transposon mutant library (Δ*pilQ*), PA4.6C and PA4.6C-R, diluted to OD_600_ in LB as described previously^49^ with the following modifications: to avoid superficial growth, a pipette was used to inoculate 1 µL of the bacterial suspension underneath 1% agar in a sterile petri dish agar plate. After 24 h, the petri dish was inverted over a waste receptacle and the agar was gently removed by an inoculation stick without touching the bacteria. The bottoms of the petri dishes were soaked in 3 mL 0.1% crystal violet stain for 10 min before gently washing twice with water, which was then quantified using ImageJ^50^. A variation of this twitching motility assay was assessed by inoculating the middle layer of an agar plate with 0.8, 1.0 and 1.2% agar and measuring the maximum diameter of the middle layer growth areas after 36 h. Swimming motility^51^ (middle of the agar plate, 0.25% agar) and swarming motility^52^ (surface of the agar plate, 0.75% agar) followed the adapted twitching motility assay described above, aside from the varied agar percentage and inoculation layer.

### Bioinformatic analysis

High-titer sterile filtered phage lysates were treated with DNAse I reaction buffer (New England Biolabs, Ipswich, MA, USA), DNAse I (~ 40 U/mL, New England Biolabs, Ipswich, MA, USA), and RNAse A (~ 0.1 mg/mL, Thermo Fisher, Waltham, MA) for 1–2 h at 37 °C prior to heat inactivation and subsequent incubation with 2X Buffer A (200 mM NaCl, 200 mM Tris, and 20 mM EDTA), proteinase K (~ 0.2 mg/mL, Thermo Fisher, Waltham, MA) and 20% (wt/vol) SDS (AmericanBio, Canton, MA, USA) for 1 h at 56 °C. Phage and bacteria genomes were obtained by Phenol–Chloroform-DNA extraction (Sigma Aldrich, Burlington, MA, USA) and isopropanol precipitation (Sigma Aldrich, Burlington, MA, USA). The Yale Center for Genome Analysis performed library preparations with the IDT EZ Kit Cat#10009821 (IDT, Skokie, IL, USA) and ran samples on the Novaseq 6000 with the sequencing reagents S4 Reagent Kit v1.5 and NovaSeq XP 4-Lane Kit v1.5 (Illumina, San Diego, CA, USA). The first contig revealed the phage genome, as confirmed by Phaster^53^ and NCBI Blast^39^. Trimmed, error-corrected and normalized reads were de novo assembled by Geneious (Geneious version 2022.2 created by Biomatters, Auckland, New Zealand). The PAP Structural Workflow v2021.02 and the PAP Functional Workflow v2022.01 of CPT Phage Galaxy was used for structural (Glimmer3, MetageneAnnotator, Sixpack) and functional (Canonical Annotation, SwissProt, NR) annotation, respectively^54^. PhageTerm was used to predict the packaging mode^55^. A phage genome map was prepared with Geneious version 2022.2 (Biomatters, Auckland, New Zealand). The most similar phage genomes were identified using NCBI Blast^39^. ABRicate and AMRFinder were used to screen for antimicrobial resistance genes in phage genomes^56,57^ (last accession date June 19, 2023). The Virulence Factor Database was used to identify potential virulence factors^58^ (last accession date June 20, 2023). The contigs of the strains PA4.6C and PA4.6C-R were checked with NCBI Blast^39^, confirming *Pseudomonas aeruginosa*. The analysis involved in determining single nucleotide polymorphism differences between PA4.6C-R (sequence read archive SRS19816778) and the parent clinical strain PA4.6C (sequence read archive SRS19816777) was performed with the Breseq pipeline^59^ using PAO1 (accession number ASM676v1) as a reference.

### Statistical analyses

All statistical analyses were performed in R Studio (version 2022.07.1 + 554, R version 4.2.1, Wilcoxon rank sum test). Data were plotted using GraphPad Prism (San Diego, CA, USA, version 9.3.1).

## Results

### Phage isolation

The first step of the optimized preparation pipeline for emergency phage therapy against *Pseudomonas aeruginosa* in our Center addresses phage isolation. Phage *vB_PaeA_SB* (hereafter abbreviated ΦSB) was environmentally sourced in New Haven, CT, USA, and was found to have lytic activity against the clinical strain PA4.6C. ΦSB was amplified on PAO1 and further processed as described (see Methods). Co-incubation of ΦSB and mid-log growth-phase PAO1 bacteria at an MOI of ~ 0.01, for 6–8 h shaking constantly at 200 rpm at 37 °C, resulted in a titer of 1e10 PFU/mL prior to purification and concentration.

### Quality tests of the primary cell banks

The second step of the pipeline entails the quality tests of the PCB. Colony picking, differential agar testing, and phenotypic morphology (detailed in Methods) ensured that strain PA4.6C was not contaminated. Strain identity and purity was confirmed by sequencing (data not shown).

## Quality tests of the primary virus stocks

### Phage identity

The third step of the pipeline relates to quality tests of the PVS beginning with phage identity. Analysis of the phage genome^39,54^ predicted dsDNA virus from the family *Autographiviridae* (43.1 kb, 62.2% GC content). The genome map of ΦSB (Fig. 1A) and the feature table (Supplementary Table S2) describe 49 open reading frames (ORFs) with 19 functionally assigned genes aside from hypothetical proteins. Structural, packaging, and scaffolding genes account for 9 of the 19 genes and 7 of the 19 genes were assigned to nucleic acid replication, recombination, regulation, and modification. PhageTerm^55^ suggested a headful packaging mechanism with a higher coverage obtained after the packaging site when aligning all raw reads (8,762,162 with 90% mapping reads) to the phage genome of ΦSB. The phages most closely related to ΦSB were phages of the genus *Phikmvviruses.* The assembled and annotated phage genome is deposited under the NCBI accession number OR208619. No virulence or lysogeny factors were identified.

### Phage characterization

The quality tests of the PVS also include a thorough phage characterization. ΦSB plaque morphology in PAO1 and PA4.6C showed uniform plaques with halos in 0.75% top layer LB agar (Supplementary Fig. S1A,B). The host range of ΦSB (Supplementary Table S1) displayed clear plaque formation spanning over 28.6% (14/49) of clinical *Pseudomonas aeruginosa* (PSA) strains isolated from sputum. Additionally, clear plaques were seen on 2/3 of non-pulmonary clinical PSA strains. Turbid plaques were observed for 17.3% (9/52) of all clinical PSA strains. Six clinical *E. coli* strains tested (negative controls) did not reveal plaques after spotting ΦSB.

**Figure 2:**
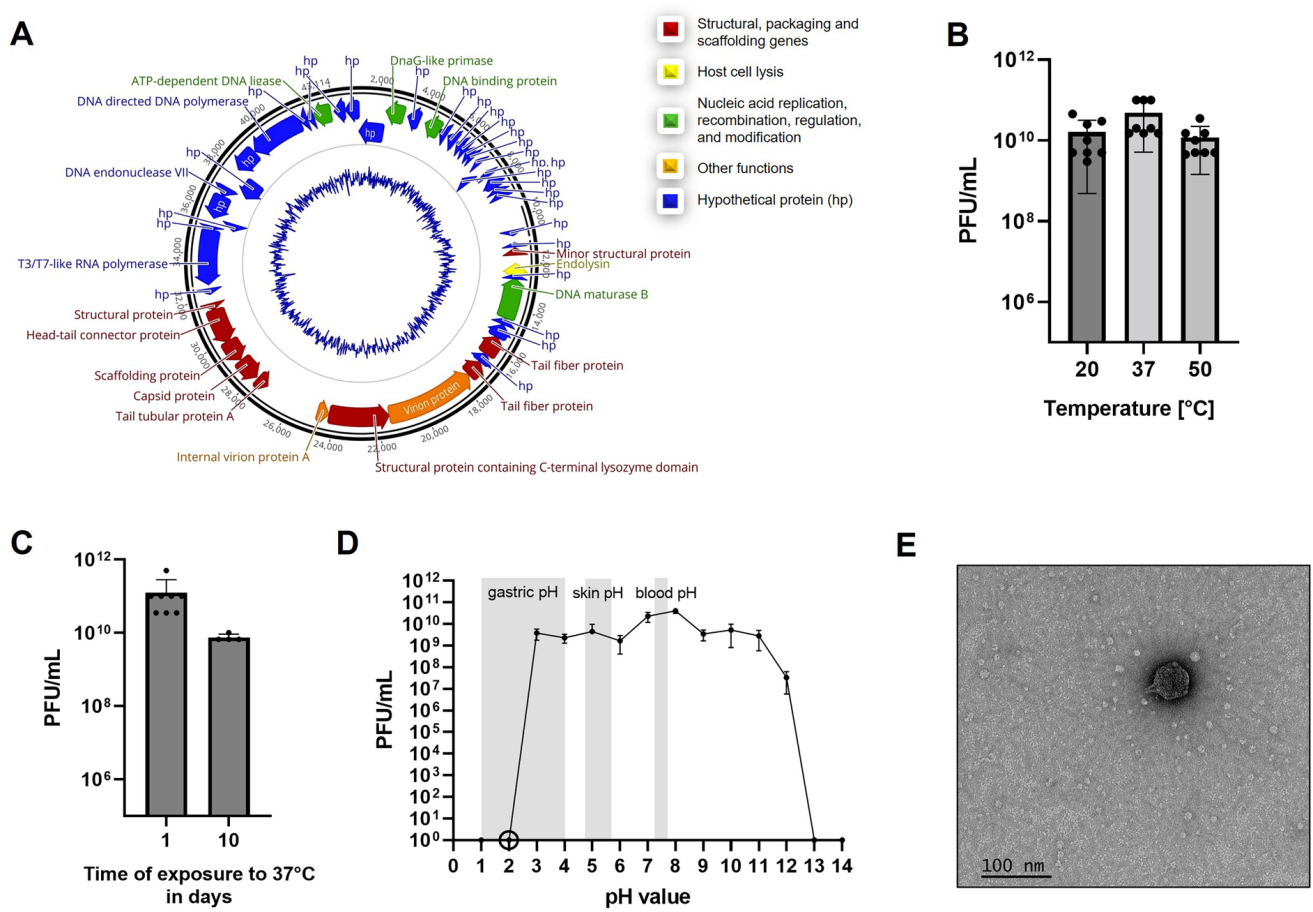
Characterization of phage ΦSB. (**A**) Inferred genome map of phage ΦSB with putative gene predictions. (**B**) Thermal stability of ΦSB for 1 h (n = 8), initial phage concentration 1e11 PFU/mL. (**C**) Thermal stability of ΦSB for 1 day (n = 8) and 10 days (n = 4) at 37 °C, initial phage concentration 1e11 PFU/mL. (**D**) pH stability of ΦSB for 24 h (n = 3), initial phage concentration 1e10 PFU/mL. 10^0^ means below the detection limit. The circle at pH 2 refers to a 2-h test at pH 2, n = 3. (**E**) TEM of ΦSB (scale bar: 100 nm).

To study phage stability, ΦSB was stored in PBS supplemented with 1 M MgSO_4_ at 2e11 PFU/mL at 4 °C for four months, and separately for seven days in 0.9% saline at 1e9 PFU/mL at 4 °C without evidence of a significant decrease in viral titer (Supplementary Fig. S2). Thermal stability is presented in Fig. 2B,C. The effect of pH was studied for 24 h (Fig. 2D) with evidence that pH < 3 or pH > 11 decreased phage titer below detection limit (Fig. 2D), which was also observed for a 2-h exposure at pH 2 (Fig. 2D). Phage titers decreased 2–3.5 logs in serum and plasma at 37 °C (Supplementary Fig. S3). There were no differences between LDH in cell culture supernatants after phage incubation compared to controls (data not shown).TEM (Fig. 2E) indicated *Autographiviridae* morphology for ΦSB^60^.

**Figure 3:**
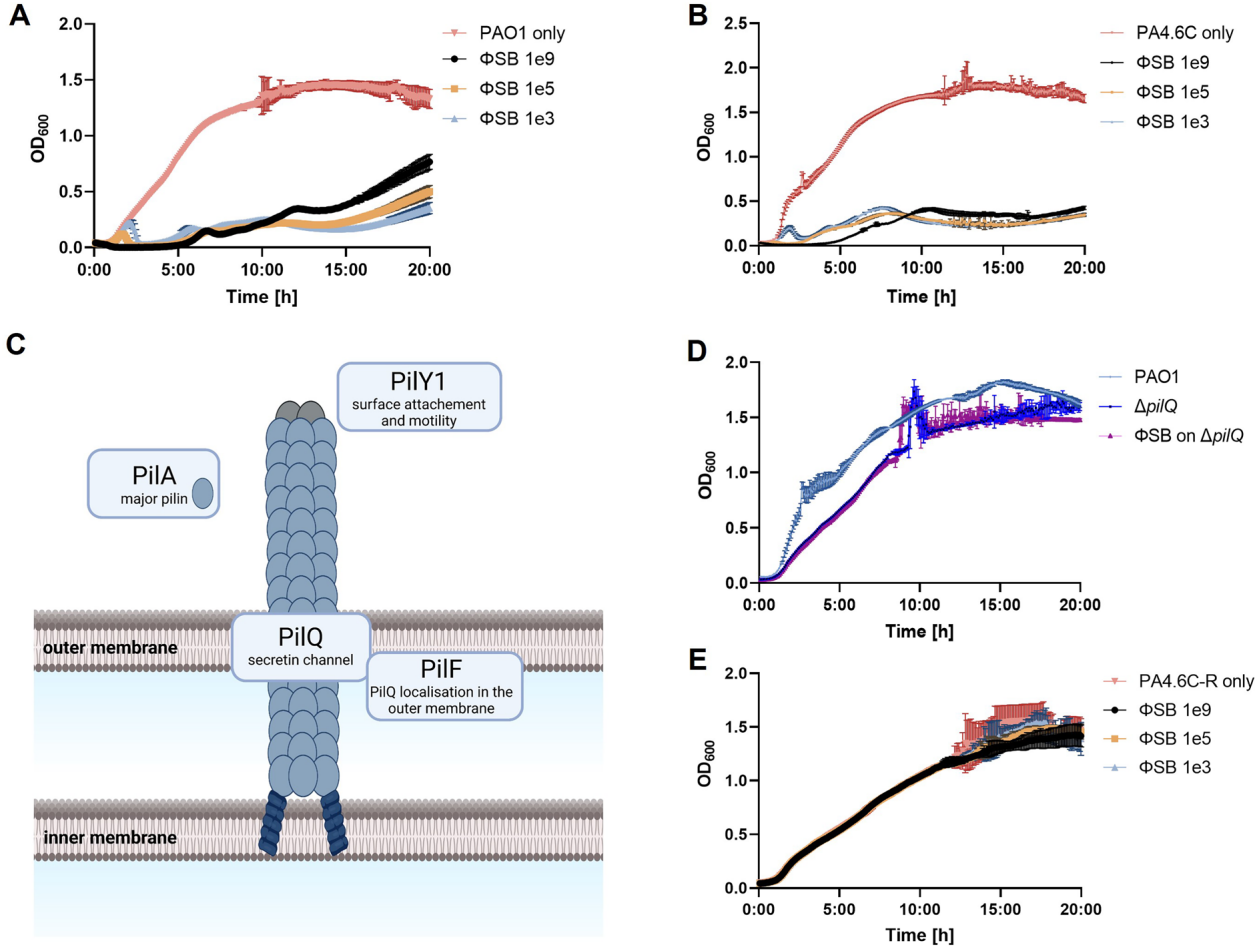
Growth kinetic changes. (**A**) Growth curve of PAO1 with ΦSB at MOI ~ 1000 (1e9 PFU/mL), ~ 0.1 (1e5 PFU/mL) and ~ 0.001 (1e3 PFU/mL), n = 6. (**B**) Growth curve of PA4.6C with ΦSB at MOI ~ 30 (1e9 PFU/mL), ~ 0.003 (1e5 PFU/mL) and ~ 0.00003 (1e3 PFU/mL), n = 6. (**C**) Simplified schematic illustration of the type-IV pili and the pilus genes tested in this study. Created with Biorender.com and based on^61–64^. (**D**) Growth curve of PAO1 and knockout strain Δ*pilQ* with ΦSB at MOI ~ 1000 (1e9 PFU/mL), n = 4–6. (**E**) Growth curve of PA4.6C-R with ΦSB at MOI ~ 700 (1e9 PFU/mL), ~ 0.07 (1e5 PFU/mL) and ~ 0.0007 (1e3 PFU/mL), n = 6.

To study suppression of PSA growth, ΦSB was added to PSA cultures at a concentration of 1e9, 1e5, and 1e3 PFU/mL for 20 h. ΦSB decreased growth of PAO1 (Fig. 3A) and PA4.6C (Fig. 3B) compared to bacteria alone.

Screening of transposon knockout strains revealed phage resistance in pilus-related knockout strains (*ΔpilA, ΔpilY1, ΔpilQ, ΔpilF*, Fig. 3C^61–64^, by spot assay. No phage amplification was observed within the incubation period, strongly suggesting that phage infection is impeded when a gene contributing to the construction of type-IV pili (TIVP) is knocked out. To examine this more closely, resistance towards the *ΔpilQ*-strain was confirmed by co-incubation of *ΔpilQ*-strain with ΦSB for 20 h (Fig. 3D). Efficiency of plating (EOP), which is the plating ability for a phage on the mutant strain relative to its plating ability on an ancestral phage sensitive host, could not be calculated because no visible plaques were produced on mutant strain *ΔpilQ* at 1e12 PFU/mL.

Adsorption assays (Supplementary Fig. S4) showed evidence for ΦSB adsorption to host cells within 10 min and the one step growth curve (Supplementary Fig. S5) showed no discernable latent period, which prevented exact burst size calculation. Within 1 h and after initial MOI ~ 0.01, phage titer increased in the one step growth curve by an average of 3.77e2 ± 4.70e2 standard deviation.

To assess the potential capability to inhibit biofilm formation and to disrupt biofilms, crystal violet staining and CFU counts of biofilm assays revealed no viable bacteria in the biofilm formation assay and a significant reduction of pre-formed biofilm by both single phage treatments observed by crystal violet staining (*p* < 0.005, Supplementary Fig. S6A) and CFU (*p* < 0.005, Supplementary Fig. S6B).

### Virulence of the phage-resistant mutant strain

The third step of the pipeline concludes with the virulence of the phage-resistant mutant strain. Phage-bacterial co-incubation was used to isolate an evolved phage-resistant mutant of PA4.6C, which identify as PA4.6C-R. EOP could not be calculated because no plaques were obtained on PA4.6C-R at 1e12 PFU/mL, which lead to no bacterial growth inhibition when adding ΦSB in different concentrations (Fig. 3E). Biofilm formation was not inhibited by the addition of ΦSB (1e9 PFU/mL) to PA4.6C-R (Supplementary Fig. S7A and B), which confirms PA4.6C-R resistance to ΦSB.

Genomic comparison between PA4.6C and PA4.6C-R (Supplementary Table S3) revealed 13 non-synonymous point mutations potentially affecting virulence: four point mutations were identified in the type-IV pili (TIVP) *pilQ* gene, which is important for TIVP biogenesis by building a secretin channel for pili extrusion^63^; one point mutation was identified in the TIVP prepilin peptidase/methyltransferase PilD; eight point mutations were identified in the gene for fucose‑binding lectin LecB, an outer membrane protein potentially involved in pathogenicity^65,66^.

**Figure 4:**
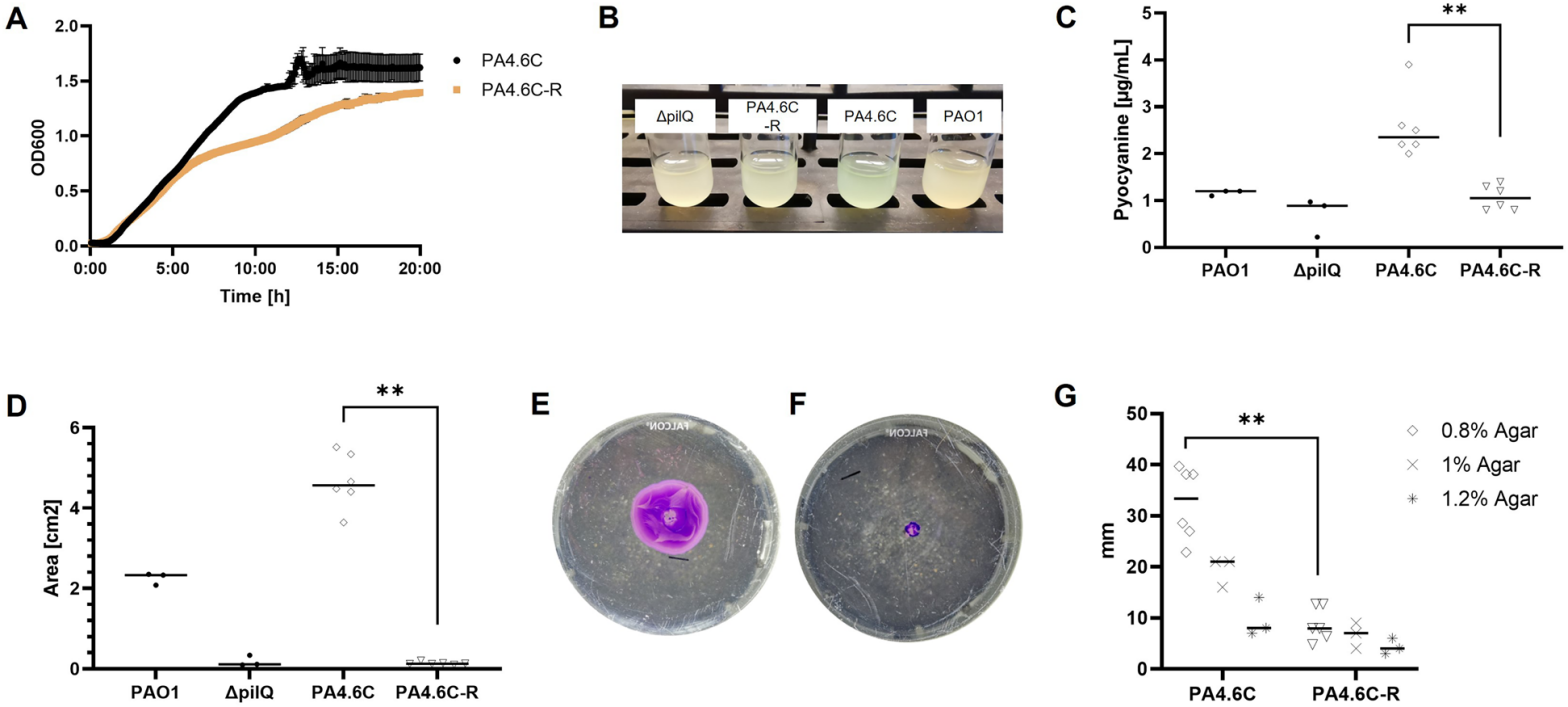
Evaluation of virulence of the phage-resistant mutant. (**A**) Growth kinetics of PA4.6C and PA4.6C-R, n = 6 wells. (**B**) Liquid cultures of knockout strain Δ*pilQ*, PA4.6C-R, PA4.6C, and PAO1. (**C)** Pyocyanine quantification (n = 3–6). (**D**) Twitching motility (n = 3). (**E**) Example petri dish of twitching motility for PA4.6C and (**F)** PA4.6C-R. (**G**) Adapted motility protocol (n = 3 and n = 6 for 0.8% agar).

The growth rate of PA4.6C-R was 40.1% compared to PA4.6C (0.50 ± 0.06 for PA4.6C and 0.32 ± 0.01 for PA4.6CR, *p* < 0.005, Fig. 4A). The area under the growth curve (AUC) decreased by 22.0% (79,662.4 ± 3100.3 for PA4.6C and 62,168.9 ± 863.9 for PA4.6C-R, *p* < 0.005, Fig. 4A). Biofilm formation was reduced in PA4.6C-R compared to the ancestral strain (Supplementary Fig. S8A,B) but no differences were found in antibiotic susceptibility testing between strains (Supplementary Fig. S9A,B). THP-1 macrophages expressed similar amounts of IL-8 after stimulation with supernatants from PA4.6C and PA4.6C-R, which were both in the range of the negative controls (Supplementary Fig. S10), and there were no differences in LDH (data not shown), a marker of cellular toxicity.

The following experiments were conducted as an attempt to account for changes in growth rate (normalization to 1e8 CFU/mL for 1:100 dilution of OD_600_ of 0.8 for PA4.6C and 1.0 for PA4.6C-R). Liquid cultures of PA4.6C were visually more blue-green compared to PA4.6C-R. Pyocyanin, a redox-active phenazine pigment responsible for the blue-green color of PSA, is involved in quorum sensing and virulence^67^. Quantification of pyocyanin revealed a decrease in pyocyanin production (58.4% decrease on average comparing PA4.6C to PA4.6C-R, *p* < 0.005, Fig. 4B,C). Elastase, an extracellular protease of PSA, has been described to facilitate tissue destruction and thereby host invasion^68,69^. Elastase production was low in both the ancestral and mutant strains (both < 0.079U/mL). Twitching motility assays presented a clearly visible, and quantifiable, decrease in motility for the mutant strain PA4.6C-R in the standard twitching motility assay (*p* < 0.005), and for the adapted twitching motility assay (0.8% agar, *p* < 0.005, see Fig. 4D-G). Swimming and swarming motility were not significantly different between PA4.6C and PA4.6C-R (Supplementary Fig. S11A,B).

### Amplification

The fourth step of the preparation pipeline for emergency phage therapy pertains to amplification. Amplification for the final phage preparation was performed with a total of 1L of ~ 0.01 MOI of the PVS with PAO1 in Tryptic Soy Broth of non-animal origin. Amplification and subsequent dialysis in PBS supplemented with 1 M MgSO_4_ resulted in a final, sterile-filtered, visually clear, and transparent 10 mL solution of ΦSB with a titer of 2e11 PFU/mL, which was found to be stable for at least 3 months at 4 °C. This solution was thus ready for dilution to the appropriate titer and buffer (e.g., saline for intravenous administration, PBS for inhalation, or for preparation with a formulation such as an ointment with an appropriate pH for respective applications). This sample would be sent for external endotoxin testing and USP < 71 > sterility for potential use for phage therapy (data not shown).

## Discussion

The emergence of antibiotic resistance, with the associated worldwide morbidity and mortality, has renewed interest in phage therapy. Despite Felix d’Hérelle’s first publication on the clinical use of phage therapy in 1921^3,7–9^, over 100 years later phage therapy is only available in the U.S. by investigational new drug (IND) application approval, which is typically a single patient IND (SPIND) reviewed by the Food and Drug Administration (FDA) under the FDA’s expanded access IND (eaIND) program. In other countries, access to phage therapy may be regulated, for example, by a temporary use authorization (in France by the French National Agency for Medicines and Health Products Safety) or by special access schemes (Therapeutic Goods Administration) in Australia^17^.

This study uses a phage targeting PSA to provide an example of the modular approach used at Yale’s Center for Phage Biology & Therapy to evaluate and prepare a phage for phage therapy for a SPIND “compassionate case”. In addition to ensuring that phage preparations meet safety and efficacy requirements, a biological understanding of phage-driven bacterial evolution is important to effectively complete such requirements. Previously published protocols or pipelines for phage therapy preparation have detailed individual steps that focused on options for phage amplification^70^, purification^37^, design of phage cocktails^71,72^, quality and safety controls for personalized phage products^73^, testing of phages within a phage bank^74^, and cGMP production guidelines^75^. Building upon this experience, this study focuses on phage isolation, characterization, PCB and PVS production, sterility, potential trade-offs of phage-resistant mutants, and phage amplification to have a product that is ready for phage therapy.

A prior publication reported that phage production requires 18.5 to 20.5 days from phage isolation to final phage solution without three times plaque picking, phage characterization, analysis of a phage-resistant mutant, or sterility testing^37^. In Belgium, phage preparation, which may be the most standardized for phage therapy in the world, includes special accreditation for pharmacies that reportedly takes ≥ 2 weeks for full characterization that results in phage designated as an Active Pharmaceutical Ingredient^24,76,77^. A pipeline set up in Germany during the peak of the COVID-19 pandemic required 2 weeks for phage isolation to lead to phage production in an innovative cell-free phage production platform^78^. However, these studies^37,78^ did not require sterility testing. In the U.S. “gold standard” sterility testing is USP < 71 > , which typically requires 14 days^79^, while future new methods may become even more rapid^40,73,80^. In addition, these protocols did not include testing for phage induced “trade-offs” or “trade-ups”^22^. It is possible to create a more time-efficient platform by completing genomic analyses, phage characterization, and testing for “trade-offs” or “trade-ups” concurrently with sterility testing. In our experience, this overlap allows completion of the pipeline in less than 3 weeks, and in some emergent cases more rapidly. Ideally, this efficiency continues to improve, especially for emergent phage therapy cases. In our experience, the U.S. FDA has been incredibly helpful by providing very timely responses to SPIND applications.

One important consideration for phage therapy that affects production is the choice of an empiric vs. targeted approach. An empiric, pre-made approach may allow for faster production but is limited by potential lack of efficacy against the target bacteria if phage sensitivity screening is not included in the pipeline^24,37,71,74,78^. To improve the production time of the targeted approach, a strategy has been proposed to develop large collections of fully characterized and ready-to-use phages for phage therapy (aka phage banks)^17,81,82^. Yerushalmy et al.^81^ previously compiled an overview of worldwide phage banks including some banks possibly suitable for human use in the future after characterization and purification of their phages [e.g., American Type Culture Collection (ATCC) or the German Collection of Microorganisms and Cell Cultures (DSMZ)]. The Israeli phage bank, with 1/5 of the phages characterized by genome sequencing, is well equipped for phage therapy^81^. Given the growing interest in phage therapy, the number of phage banks will likely increase. The phage bank approach requires monitoring for phage titer(s) and sterility, which will require continual testing and updating^71^. Our experience^83^, and others^74^, suggests that such characterization, monitoring, and re-amplification provides phages that target relevant clinical pathogens. For some bacteria where phages have high host specificity, such a library may require many phages to maintain clinical relevance. An example is *Klebsiella pneumoniae*, which has been predicted to require > 500 phages^78,84–86^. Continued efforts are required to maintain a diverse phage library for clinically relevant bacteria, which will be facilitated by collaborations within the phage community.

The pipeline reported here includes additional phage characterization such as investigating the phage receptor. This can assist to increase sophistication of phage cocktail(s)^71,87^, but to date has not been addressed in prior phage therapy pipelines^37,70,73,74^. In our study, we used spot-based screening of a knock-out library, phage resistance to Δ*pilQ* in liquid culture, and mutations found in PA4.6C-R that suggested ΦSB adheres to Type-IV pili (TIVP), which are common receptors for PSA phages^62,63^. However, we did not confirm this finding by complementation of TIVP^88^. While the consequences of all non-synonymous mutations between phage-resistant mutant strain and phage-sensitive parent strain are not entirely understood, they are sufficient to both cause phage resistance and—regarding *pilQ*—a trade-off similar to a transposon knockout mutant. Therefore, while all possible effects of these mutations are beyond the scope of this pipeline manuscript, the outcome is consistent with disruption of TIVP function. Although not directly pertinent to emergency phage preparation, we are actively investigating the specific roles of each mutation as part of our ongoing laboratory projects.

Further phage characterization in this pipeline includes clinically important information such as phage growth kinetics at different titers, pH and thermal stability, and stability in saline. As noted by Gelman et al.^74^, we agree that phage characterization can be reduced to meet time limits of patients: electron microscopy, host range testing, adsorption assay, and the one-step-growth curves are not essential information for phage therapy in individual patients. In addition, the clearance capability of the phage could be roughly estimated by growth kinetics instead of short latent periods or large burst sizes^71^. In this study, ΦSB suppressed PSA growth at a low concentration of 1e3 PFU/mL in liquid co-incubation. Specific experiments tailored to the possible clinical application included biofilm inhibition and disruption assays in this study. Gelman et al.^74^ recommended characterizing biofilm inhibition by crystal violet staining, confocal microscopy, and electron microscopy. We propose to also use an assay indicating cell viability by CFUs. To efficiently target multiple PSA bacteria in patients with a single phage, we recommend conducting a comprehensive EOP analysis across a broad spectrum of host bacteria.

Another consideration for orthopedic treatments is more in-depth biofilm assays that use human bone material or metal implants, by varying MOIs, and by pre-forming biofilm for various timepoints before adding phages. In preparation for i.v. treatments, phage titer can be determined after 30- and 60-min incubation with the patient serum as described in the Belgian Standardized Multidisciplinary Treatment Protocol by Onsea et al.^77^. For certain bacterial infections, particularly polymicrobial infections, phage interactions in a community context may provide useful information for treatment approaches, although these are complicated experiments to design and to execute^89^.

We argue that the phage therapy pipelines should include, at least briefly, an analysis of phage-resistant bacteria that results from evolutionary selection pressure by phages. Similar to antibiotics, phages exert an evolutionary selection pressure on bacteria^3,22,33,35,71^. The resulting trade-offs or trade-ups of phage-driven escape mutant bacteria have to be considered prior to personalized use to prevent treatment with a phage that might foster the generation of more virulent bacteria^3,22,35^. Our phage therapy approach takes advantage of PSA evolved resistance by choosing phages that target bacterial cell surface receptors that contribute to antibiotic resistance or virulence^3,34,35,83^. In addition to killing PSA, this strategy directs evolutionary selection toward trade-offs that compromise bacterial virulence in surviving bacterial mutants^35^. However, the potential for increased virulence, or trade-up, exists. Therefore, we studied phage-resistant mutant PA4.6C-R and found trade-offs that include changes in growth rate, pyocyanin production, motility, and biofilm formation, which suggest attenuated virulence in surviving bacteria.

Twitching motility depends on orchestrated pilus motility, which allows PSA to migrate into poorly accessible body spaces^62,90–92^. PA4.6C-R showed decreased twitching motility that is consistent with four point mutations identified in its genome in the gene coding for *pilQ*, which is a component of TIVP^62,90^. The gene of *pilD* only exhibited one point mutation, which may not affect its phenotype. The eight point mutations in the gene for fucose-specific lectin, *lecB*, might impair adherence and biofilm stability^65,66^. Other point mutations in PA4.6C-R were identified by the breseq pipeline in domains of unknown function and prophage genes. Additionally, three single point mutations were found in three genes of the two-partner secretion (Tps) systems of TpsA and TpsB, one point mutation in a gene coding for the basic-amino-acid-specific porin OprD^93^, and three single point mutations in three different genes for DNA modification. In the case of genomic analyses pointing towards increased virulence of phage-resistant mutants compared to the ancestral patient strain, we recommend reassessing the phenotypic implications and reconsidering the use of the phage in question for phage therapy. Additional assays studying the effect of PA4.6C-R to increase cytokine and chemokine production on relevant cells may help to ensure that no trade-up is present. In our experiments, this included measuring IL-8, which is an important chemokine for neutrophil recruitment.

Such a pipeline may also affect the approach to phage cocktails, whose use has been re-evaluated in the past few years^94–96^. We have learned that bacterial load reduction is not necessarily enhanced by a higher number of phages in the cocktail^96^, and that a combination of phages in a cocktail might be neutral, beneficial or detrimental to bacterial eradication^94^ with the biggest concern that phage cocktails might result in broadly phage-resistant bacteria ^74^. Thus, we recommend designing these phage cocktails according to different receptors and to take into account synergies and antagonisms by a systematic phage design approach^71,95–100^.

The pipeline presented here has several limitations. First, for PSA we have extensive knowledge about phage-bacteria interaction that may not be present for other bacteria considered for phage therapy. While we added assays for trade-offs and trade-ups, our pipeline did not include antibiotic synergy testing^101^. We have not routinely performed this test for PSA due to various multidrug- and pandrug-resistant bacteria in our phage therapy cases. The evaluation of these approaches, which also depends on the location of the infection being treated, needs to be refined in the future. Innovative methods of safe and high-titer phage production such as the cell-free phage platform were not applied in this study but are detailed in a previous phage preparation pipeline^78,102^. Pharmaceutical formulations, e.g., ointments, encapsulations for oral administration, or hydrogels are not covered here.

Future studies of phage pipelines or protocols for personalized therapy could explore specific steps required for particular phage applications, such as inhaled therapy for cystic fibrosis patients, biofilms on aortic prostheses, foot ulcers with polymicrobial infections, or phage therapy targeting the gut microbiome. All of this will be informed by an increasing number of clinical trials on phage therapy.

In summary, we present an efficient modular pipeline for individualized, timely, safe and high-quality phage solutions for SPINDs, including a thorough characterization of the phage and a representative phage-resistant mutant, allowing for rational decision for the most appropriate phage candidate(s) for each patient. A novel PSA phage is characterized, and studies are used to identify phage-induced trade-offs, while minimizing phage-induced trade-ups. Overall, phage characterization should be tailored to the specific use and time constraints of each patient.

### Supplementary Information

Supplementary Information.

## Data Availability

The datasets used and/or analyzed during the current study are available from the corresponding author on reasonable request.
