# Supplementary material for: Optimized preparation pipeline for emergency phage therapy against Pseudomonas aeruginosa at Yale University
Source: Sci Rep. 2024 Feb 1;14:2657. doi: 10.1038/s41598-024-52192-3 (PMC10834462; doi:10.1038/s41598-024-52192-3)

**Supplementary Material**

**Optimized preparation pipeline for emergency phage therapy against *Pseudomonas aeruginosa* at Yale University**

[**Supplementary Table S1:** Brief description of bacterial strains of this study and brief host range analysis. 2](#_Toc154012982)

[**Supplementary Table S2:** Feature table of the genome of phage SB. 4](#_Toc154012983)

[**Supplementary Table S3:** Summary of the Breseq output. 7](#_Toc154012984)

[**Supplementary Fig. S1:** Plaque morphology of phage SB on PAO1 (A) and PA4.6C (B). 8](#_Toc154012985)

[**Supplementary Fig. S2:** Phage stability under salt exposure. 9](#_Toc154012986)

[**Supplementary Fig. S3:** Phage stability under serum and plasma exposure. 9](#_Toc154012987)

[**Supplementary Fig. S4:** Adsorption of phage SB to host cells. 10](#_Toc154012988)

[**Supplementary Fig. S5:** One step growth curve for phage SB population on host cells. 10](#_Toc154012989)

[**Supplementary Fig. S6:** Phage SB disrupts bacterial biofilm formation. 11](#_Toc154012990)

[**Supplementary Fig. S7:** Phage SB does not alter bacterial biofilm formation of phage-resistant mutant PA4.6C-R. 11](#_Toc154012991)

[**Supplementary Fig. S8:** Comparison of biofilm formation capabilities of PA4.6C and PA4.6C-R bacteria. 12](#_Toc154012992)

[**Supplementary Fig. S9:** Comparison of antibiotic susceptibilities of PA4.6C and PA4.6C-R bacteria. 12](#_Toc154012993)

[**Supplementary Fig. S10:** PA4.6C and PA4.6C-R strains do not differentially stimulate IL-8 production by THP-1 cells. 13](#_Toc154012994)

[**Supplementary Fig. S11:** Swimming and swarming motilities of PA4.6C and PA4.6C-R do not differ. 13](#_Toc154012995)

**Supplementary Table S1:** Brief description of bacterial strains of this study and brief host range analysis. N no plaque, C clear plaque, T turbid plaque.

|  | **Strain** | **Species** | **Origin** | **Isolation** | **Host range analysis with phage SB** |
| --- | --- | --- | --- | --- | --- |
| Reference strain | PAO1 | *Pseudomonas aeruginosa* | B. Kazmierczak, Yale University, New Haven, CT, USA | Infected wound, Melbourne, Australia, 1954 | C |
|  | PAO1 transposon mutant library | *Pseudomonas aeruginosa* | C. Manoil, University of Washington, Seattle, WA, USA | Infected wound, Melbourne, Australia, 1954 | - |
| De-identified patient strains | PA4.6C | *Pseudomonas aeruginosa* | Cystic Fibrosis Foundation | LVAD driveline exit site | C |
|  | CF 1 | *Pseudomonas aeruginosa* | Cystic Fibrosis Foundation | Pulmonary infection, isolated from sputum | N |
|  | CF 2 | *Pseudomonas aeruginosa* | Cystic Fibrosis Foundation | Pulmonary infection, isolated from sputum | T |
|  | CF 3 | *Pseudomonas aeruginosa* | Cystic Fibrosis Foundation | Pulmonary infection, isolated from sputum | N |
|  | CF 4 | *Pseudomonas aeruginosa* | Cystic Fibrosis Foundation | Pulmonary infection, isolated from sputum | C |
|  | CF 5 | *Pseudomonas aeruginosa* | Cystic Fibrosis Foundation | Pulmonary infection, isolated from sputum | T |
|  | CF 6 | *Pseudomonas aeruginosa* | Cystic Fibrosis Foundation | Pulmonary infection, isolated from sputum | T |
|  | CF 7 | *Pseudomonas aeruginosa* | Cystic Fibrosis Foundation | Pulmonary infection, isolated from sputum | T |
|  | CF 8 | *Pseudomonas aeruginosa* | Cystic Fibrosis Foundation | Pulmonary infection, isolated from sputum | T |
|  | CF 9 | *Pseudomonas aeruginosa* | Cystic Fibrosis Foundation | Pulmonary infection, isolated from sputum | N |
|  | CF 10 | *Pseudomonas aeruginosa* | Cystic Fibrosis Foundation | Pulmonary infection, isolated from sputum | T |
|  | CF 11 | *Pseudomonas aeruginosa* | Cystic Fibrosis Foundation | Pulmonary infection, isolated from sputum | N |
|  | CF 12 | *Pseudomonas aeruginosa* | Cystic Fibrosis Foundation | Pulmonary infection, isolated from sputum | C |
|  | CF 13 | *Pseudomonas aeruginosa* | Cystic Fibrosis Foundation | Pulmonary infection, isolated from sputum | C |
|  | CF 14 | *Pseudomonas aeruginosa* | Cystic Fibrosis Foundation | Pulmonary infection, isolated from sputum | N |
|  | CF 15 | *Pseudomonas aeruginosa* | Cystic Fibrosis Foundation | Pulmonary infection, isolated from sputum | C |
|  | CF 16 | *Pseudomonas aeruginosa* | Cystic Fibrosis Foundation | Pulmonary infection, isolated from sputum | T |
|  | CF 17 | *Pseudomonas aeruginosa* | Cystic Fibrosis Foundation | Pulmonary infection, isolated from sputum | N |
|  | CF 19 | *Pseudomonas aeruginosa* | Cystic Fibrosis Foundation | Pulmonary infection, isolated from sputum | C |
|  | CF 20 | *Pseudomonas aeruginosa* | Cystic Fibrosis Foundation | Pulmonary infection, isolated from sputum | N |
|  | CF 21 | *Pseudomonas aeruginosa* | Cystic Fibrosis Foundation | Pulmonary infection, isolated from sputum | C |
|  | CF 22 | *Pseudomonas aeruginosa* | Cystic Fibrosis Foundation | Pulmonary infection, isolated from sputum | N |
|  | CF 23 | *Pseudomonas aeruginosa* | Cystic Fibrosis Foundation | Pulmonary infection, isolated from sputum | N |
|  | CF 24 | *Pseudomonas aeruginosa* | Cystic Fibrosis Foundation | Pulmonary infection, isolated from sputum | T |
|  | CF 25 | *Pseudomonas aeruginosa* | Cystic Fibrosis Foundation | Pulmonary infection, isolated from sputum | C |
|  | CF 26 | *Pseudomonas aeruginosa* | Cystic Fibrosis Foundation | Pulmonary infection, isolated from sputum | C |
|  | CF 27 | *Pseudomonas aeruginosa* | Cystic Fibrosis Foundation | Pulmonary infection, isolated from sputum | N |
|  | CF 28 | *Pseudomonas aeruginosa* | Cystic Fibrosis Foundation | Pulmonary infection, isolated from sputum | N |
|  | CF 29 | *Pseudomonas aeruginosa* | Cystic Fibrosis Foundation | Pulmonary infection, isolated from sputum | N |
|  | CF 30 | *Pseudomonas aeruginosa* | Cystic Fibrosis Foundation | Pulmonary infection, isolated from sputum | N |
|  | CF 31 | *Pseudomonas aeruginosa* | Cystic Fibrosis Foundation | Pulmonary infection, isolated from sputum | C |
|  | CF 32 | *Pseudomonas aeruginosa* | Cystic Fibrosis Foundation | Pulmonary infection, isolated from sputum | N |
|  | CF 33 | *Pseudomonas aeruginosa* | Cystic Fibrosis Foundation | Pulmonary infection, isolated from sputum | N |
|  | CF 34 | *Pseudomonas aeruginosa* | Cystic Fibrosis Foundation | Pulmonary infection, isolated from sputum | N |
|  | CF 35 | *Pseudomonas aeruginosa* | Cystic Fibrosis Foundation | Pulmonary infection, isolated from sputum | C |
|  | CF 36 | *Pseudomonas aeruginosa* | Cystic Fibrosis Foundation | Pulmonary infection, isolated from sputum | C |
|  | CF 37 | *Pseudomonas aeruginosa* | Cystic Fibrosis Foundation | Pulmonary infection, isolated from sputum | T |
|  | CF 38 | *Pseudomonas aeruginosa* | Cystic Fibrosis Foundation | Pulmonary infection, isolated from sputum | N |
|  | CF 39 | *Pseudomonas aeruginosa* | Cystic Fibrosis Foundation | Pulmonary infection, isolated from sputum | N |
|  | CF 40 | *Pseudomonas aeruginosa* | Cystic Fibrosis Foundation | Pulmonary infection, isolated from sputum | N |
|  | CF 41 | *Pseudomonas aeruginosa* | Cystic Fibrosis Foundation | Pulmonary infection, isolated from sputum | N |
|  | CF 42 | *Pseudomonas aeruginosa* | Cystic Fibrosis Foundation | Pulmonary infection, isolated from sputum | N |
|  | CF 43 | *Pseudomonas aeruginosa* | Cystic Fibrosis Foundation | Pulmonary infection, isolated from sputum | N |
|  | CF 44 | *Pseudomonas aeruginosa* | Cystic Fibrosis Foundation | Pulmonary infection, isolated from sputum | C |
|  | CF 45 | *Pseudomonas aeruginosa* | Cystic Fibrosis Foundation | Pulmonary infection, isolated from sputum | C |
|  | CF 46 | *Pseudomonas aeruginosa* | Cystic Fibrosis Foundation | Pulmonary infection, isolated from sputum | N |
|  | CF 47 | *Pseudomonas aeruginosa* | Cystic Fibrosis Foundation | Pulmonary infection, isolated from sputum | N |
|  | CF 48 | *Pseudomonas aeruginosa* | Cystic Fibrosis Foundation | Pulmonary infection, isolated from sputum | N |
|  | CF 49 | *Pseudomonas aeruginosa* | Cystic Fibrosis Foundation | Pulmonary infection, isolated from sputum | C |
|  | CF 50 | *Pseudomonas aeruginosa* | Cystic Fibrosis Foundation | Pulmonary infection, isolated from sputum | N |
|  | PA 74 | *Pseudomonas aeruginosa* | Yale New Haven Hospital, New Haven, CT, USA | Pyelonephritis | N |
|  | PA D8 | *Pseudomonas aeruginosa* | Hershey Medical Center, Hershey, PA, USA | Necrotizing pancreatitis | C |
|  | PA SC3 | *Pseudomonas aeruginosa* | University of California San Diego, La Jolla, CA, USA | Femoral graft infection | C |
|  | EC D35 | *Escherichia coli* | Yale New Haven Hospital, New Haven, CT, USA | Urinary tract infection | N |
|  | EC 8.23 | *Escherichia coli* | Mayo Clinic, Rochester, MN, USA | Urinary tract infection | N |
|  | EC D39 | *Escherichia coli* | Yale New Haven Hospital, New Haven, CT, USA | Urinary tract infection | N |
|  | EC R91 | *Escherichia coli* | Yale New Haven Hospital, New Haven, CT, USA | Urinary tract infection | N |
|  | EC B6 | *Escherichia coli* | Yale New Haven Hospital, New Haven, CT, USA | Urinary tract infection | N |
|  | EC 22 | *Escherichia coli* | Yale New Haven Hospital, New Haven, CT, USA | Urinary tract infection | N |

# **Supplementary Table S2:** Feature table of the genome of phage SB.

| **Name** | **Type** | **Minimum** | **Maximum** | **Length** | **Direction** |
| --- | --- | --- | --- | --- | --- |
| regulatory | regulatory | 42980 | 42982 | 3 | reverse |
| regulatory | regulatory | 42190 | 42193 | 4 | reverse |
| putative ATP-dependent DNA ligase CDS | CDS | 42179 | 42970 | 792 | reverse |
| gene | gene | 42179 | 42982 | 804 | reverse |
| regulatory | regulatory | 41909 | 41913 | 5 | reverse |
| hypothetical protein CDS | CDS | 41898 | 42182 | 285 | reverse |
| gene | gene | 41898 | 42193 | 296 | reverse |
| regulatory | regulatory | 41577 | 41579 | 3 | reverse |
| hypothetical protein CDS | CDS | 41566 | 41895 | 330 | reverse |
| gene | gene | 41566 | 41913 | 348 | reverse |
| putative DNA directed DNA polymerase CDS | CDS | 39140 | 41569 | 2430 | reverse |
| gene | gene | 39140 | 41579 | 2440 | reverse |
| regulatory | regulatory | 39092 | 39094 | 3 | reverse |
| regulatory | regulatory | 38039 | 38042 | 4 | reverse |
| hypothetical protein CDS | CDS | 38035 | 39084 | 1050 | reverse |
| gene | gene | 38035 | 39094 | 1060 | reverse |
| regulatory | regulatory | 37115 | 37119 | 5 | reverse |
| hypothetical protein CDS | CDS | 37094 | 38035 | 942 | reverse |
| gene | gene | 37094 | 38042 | 949 | reverse |
| regulatory | regulatory | 36672 | 36675 | 4 | reverse |
| putative DNA endonuclease VII CDS | CDS | 36664 | 37104 | 441 | reverse |
| gene | gene | 36664 | 37119 | 456 | reverse |
| hypothetical protein CDS | CDS | 35621 | 36667 | 1047 | reverse |
| gene | gene | 35621 | 36675 | 1055 | reverse |
| regulatory | regulatory | 35615 | 35621 | 7 | reverse |
| regulatory | regulatory | 35255 | 35258 | 4 | reverse |
| hypothetical protein CDS | CDS | 35237 | 35611 | 375 | reverse |
| gene | gene | 35237 | 35621 | 385 | reverse |
| hypothetical protein CDS | CDS | 35083 | 35247 | 165 | reverse |
| gene | gene | 35083 | 35258 | 176 | reverse |
| regulatory | regulatory | 35080 | 35084 | 5 | reverse |
| putative RNA polymerase CDS | CDS | 32627 | 35074 | 2448 | reverse |
| gene | gene | 32627 | 35084 | 2458 | reverse |
| regulatory | regulatory | 32463 | 32467 | 5 | reverse |
| hypothetical protein CDS | CDS | 32202 | 32453 | 252 | reverse |
| gene | gene | 32202 | 32467 | 266 | reverse |
| regulatory | regulatory | 31796 | 31799 | 4 | reverse |
| putative structural protein CDS | CDS | 31488 | 31784 | 297 | reverse |
| gene | gene | 31488 | 31799 | 312 | reverse |
| regulatory | regulatory | 31482 | 31486 | 5 | reverse |
| regulatory | regulatory | 29949 | 29953 | 5 | reverse |
| putative head-tail connector protein CDS | CDS | 29944 | 31476 | 1533 | reverse |
| gene | gene | 29944 | 31486 | 1543 | reverse |
| putative scaffolding protein CDS | CDS | 28972 | 29940 | 969 | reverse |
| gene | gene | 28972 | 29953 | 982 | reverse |
| regulatory | regulatory | 28926 | 28928 | 3 | reverse |
| putative capsid protein CDS | CDS | 27912 | 28919 | 1008 | reverse |
| gene | gene | 27912 | 28928 | 1017 | reverse |
| regulatory | regulatory | 27823 | 27827 | 5 | reverse |
| putative tail tubular protein A CDS | CDS | 27261 | 27815 | 555 | reverse |
| gene | gene | 27261 | 27827 | 567 | reverse |
| regulatory | regulatory | 24785 | 24789 | 5 | reverse |
| regulatory | regulatory | 24240 | 24245 | 6 | reverse |
| putative internal virion protein A CDS | CDS | 24233 | 24781 | 549 | reverse |
| gene | gene | 24233 | 24789 | 557 | reverse |
| regulatory | regulatory | 21540 | 21543 | 4 | reverse |
| putative structural protein CDS | CDS | 21537 | 24233 | 2697 | reverse |
| gene | gene | 21537 | 24245 | 2709 | reverse |
| regulatory | regulatory | 17525 | 17528 | 4 | reverse |
| putative virion protein CDS | CDS | 17520 | 21533 | 4014 | reverse |
| gene | gene | 17520 | 21543 | 4024 | reverse |
| regulatory | regulatory | 16771 | 16775 | 5 | reverse |
| putative tail fiber protein CDS | CDS | 16763 | 17518 | 756 | reverse |
| gene | gene | 16763 | 17528 | 766 | reverse |
| regulatory | regulatory | 16321 | 16323 | 3 | reverse |
| hypothetical protein CDS | CDS | 16305 | 16763 | 459 | reverse |
| gene | gene | 16305 | 16775 | 471 | reverse |
| regulatory | regulatory | 15410 | 15413 | 4 | reverse |
| putative tail fiber protein CDS | CDS | 15404 | 16312 | 909 | reverse |
| gene | gene | 15404 | 16323 | 920 | reverse |
| regulatory | regulatory | 14805 | 14808 | 4 | reverse |
| hypothetical protein CDS | CDS | 14795 | 15400 | 606 | reverse |
| gene | gene | 14795 | 15413 | 619 | reverse |
| hypothetical protein CDS | CDS | 14490 | 14795 | 306 | reverse |
| gene | gene | 14490 | 14808 | 319 | reverse |
| regulatory | regulatory | 14485 | 14490 | 6 | reverse |
| regulatory | regulatory | 12687 | 12690 | 4 | reverse |
| putative DNA maturase B CDS | CDS | 12675 | 14480 | 1806 | reverse |
| gene | gene | 12675 | 14490 | 1816 | reverse |
| regulatory | regulatory | 12489 | 12494 | 6 | reverse |
| hypothetical protein CDS | CDS | 12478 | 12678 | 201 | reverse |
| gene | gene | 12478 | 12690 | 213 | reverse |
| putative endolysin CDS | CDS | 11999 | 12481 | 483 | reverse |
| gene | gene | 11999 | 12494 | 496 | reverse |
| regulatory | regulatory | 11629 | 11633 | 5 | reverse |
| putative minor structural protein CDS | CDS | 11308 | 11622 | 315 | reverse |
| gene | gene | 11308 | 11633 | 326 | reverse |
| regulatory | regulatory | 11268 | 11271 | 4 | reverse |
| hypothetical protein CDS | CDS | 11065 | 11259 | 195 | reverse |
| gene | gene | 11065 | 11271 | 207 | reverse |
| hypothetical protein CDS | CDS | 10547 | 10759 | 213 | forward |
| gene | gene | 10535 | 10759 | 225 | forward |
| regulatory | regulatory | 10535 | 10539 | 5 | forward |
| regulatory | regulatory | 9008 | 9011 | 4 | reverse |
| regulatory | regulatory | 8723 | 8726 | 4 | reverse |
| hypothetical protein CDS | CDS | 8717 | 9001 | 285 | reverse |
| gene | gene | 8717 | 9011 | 295 | reverse |
| hypothetical protein CDS | CDS | 8490 | 8717 | 228 | reverse |
| gene | gene | 8490 | 8726 | 237 | reverse |
| regulatory | regulatory | 8487 | 8491 | 5 | reverse |
| hypothetical protein CDS | CDS | 7940 | 8479 | 540 | reverse |
| gene | gene | 7940 | 8491 | 552 | reverse |
| regulatory | regulatory | 7885 | 7888 | 4 | reverse |
| regulatory | regulatory | 7780 | 7782 | 3 | reverse |
| hypothetical protein CDS | CDS | 7773 | 7877 | 105 | reverse |
| gene | gene | 7773 | 7888 | 116 | reverse |
| hypothetical protein CDS | CDS | 7651 | 7776 | 126 | reverse |
| gene | gene | 7651 | 7782 | 132 | reverse |
| regulatory | regulatory | 7582 | 7584 | 3 | reverse |
| regulatory | regulatory | 7228 | 7231 | 4 | reverse |
| hypothetical protein CDS | CDS | 7204 | 7572 | 369 | reverse |
| gene | gene | 7204 | 7584 | 381 | reverse |
| hypothetical protein CDS | CDS | 6990 | 7217 | 228 | reverse |
| gene | gene | 6990 | 7231 | 242 | reverse |
| regulatory | regulatory | 6821 | 6824 | 4 | reverse |
| regulatory | regulatory | 6640 | 6645 | 6 | reverse |
| hypothetical protein CDS | CDS | 6632 | 6811 | 180 | reverse |
| gene | gene | 6632 | 6824 | 193 | reverse |
| regulatory | regulatory | 6356 | 6359 | 4 | reverse |
| hypothetical protein CDS | CDS | 6345 | 6632 | 288 | reverse |
| gene | gene | 6345 | 6645 | 301 | reverse |
| regulatory | regulatory | 6107 | 6111 | 5 | reverse |
| hypothetical protein CDS | CDS | 6097 | 6348 | 252 | reverse |
| gene | gene | 6097 | 6359 | 263 | reverse |
| hypothetical protein CDS | CDS | 5807 | 6100 | 294 | reverse |
| gene | gene | 5807 | 6111 | 305 | reverse |
| regulatory | regulatory | 5736 | 5740 | 5 | reverse |
| hypothetical protein CDS | CDS | 5309 | 5728 | 420 | reverse |
| gene | gene | 5309 | 5740 | 432 | reverse |
| regulatory | regulatory | 5248 | 5253 | 6 | reverse |
| regulatory | regulatory | 4886 | 4890 | 5 | reverse |
| hypothetical protein CDS | CDS | 4881 | 5240 | 360 | reverse |
| gene | gene | 4881 | 5253 | 373 | reverse |
| putative DNA binding protein CDS | CDS | 4150 | 4878 | 729 | reverse |
| gene | gene | 4150 | 4890 | 741 | reverse |
| regulatory | regulatory | 3891 | 3894 | 4 | reverse |
| hypothetical protein CDS | CDS | 3341 | 3880 | 540 | reverse |
| gene | gene | 3341 | 3894 | 554 | reverse |
| regulatory | regulatory | 3164 | 3168 | 5 | reverse |
| regulatory | regulatory | 2371 | 2375 | 5 | reverse |
| putative DnaG-like primase CDS | CDS | 2331 | 3155 | 825 | reverse |
| gene | gene | 2331 | 3168 | 838 | reverse |
| regulatory | regulatory | 1115 | 1117 | 3 | reverse |
| hypothetical protein CDS | CDS | 1094 | 2362 | 1269 | reverse |
| gene | gene | 1094 | 2375 | 1282 | reverse |
| hypothetical protein CDS | CDS | 484 | 1104 | 621 | reverse |
| gene | gene | 484 | 1117 | 634 | reverse |

**Supplementary Table S3:** Summary of the Breseq output. Identification of predicted mutations distinguishing PA4.6C-R from the parent clinical strain PA4.6C.

| **position** | **mutation** | **annotation** | **gene** | **description** |
| --- | --- | --- | --- | --- |
| 4,724,689 | G→T | Q52K (CAG→AAG) | HKX07_RS21845 ← | major capsid protein |
| 4,724,693 | A→C | D50E (GAT→GAG) | HKX07_RS21845 ← | major capsid protein |
| 4,726,443 | C→T | R9H (CGC→CAC) | HKX07_RS21860 ← | DUF5447 family protein |
| 290,976 | G→T | T10K (ACA→AAA) | HKX07_RS01325 ← | DUF2875 family protein |
| 5,014,175 | A→G | T18A (ACC→GCC) | pilD → | type IV prepilin peptidase/methyltransferase PilD |
| 164,556 | T→C | T172A (ACC→GCC) | HKX07_RS00755 ← | nucleoside 2‑deoxyribosyltransferase |
| 1,742,600 | T→A | Q67L (CAG→CTG) | lecB ← | fucose‑binding lectin LecB |
| 1,742,604 | C→T | V66I (GTC→ATC) | lecB ← | fucose‑binding lectin LecB |
| 1,742,619 | T→C | S61G (AGT→GGC) | lecB ← | fucose‑binding lectin LecB |
| 1,742,622 | T→C | S60G (AGC→GGC) | lecB ← | fucose‑binding lectin LecB |
| 1,742,640 | G→T | Q54K (CAG→AAG) | lecB ← | fucose‑binding lectin LecB |
| 1,742,642 | G→C | T53S (ACC→AGC) | lecB ← | fucose‑binding lectin LecB |
| 1,742,652 | C→T | V50I (GTC→ATC) | lecB ← | fucose‑binding lectin LecB |
| 1,742,675 | C→G | S42T (AGC→ACC) | lecB ← | fucose‑binding lectin LecB |
| 698,334 | A→C | M1085L (ATG→CTC) | HKX07_RS03275 → | phage tail protein |
| 698,336 | G→C | M1085L (ATG→CTC) | HKX07_RS03275 → | phage tail protein |
| 43,932 | T→C | V340A (GTG→GCG) | tpsA2 → | two‑partner secretion system putative hemagglutinin TpsA2 |
| 4,726,389 | A→G | V27A (GTC→GCC) | HKX07_RS21860 ← | DUF5447 family protein |
| 5,629,680 | G→C | I558M (ATC→ATG) | pilQ ← | type 4a pilus secretin PilQ |
| 328,578 | G→C | S432T (AGC→ACC) | HKX07_RS01485 → | OprD family porin |
| 4,272,849 | T→A | L47Q (CTG→CAG) | HKX07_RS19625 → | DUF2195 family protein |
| 5,629,286 | T→A | T690S (ACC→TCC) | pilQ ← | type 4a pilus secretin PilQ |
| 628,445 | G→A | L64F (CTC→TTC) | HKX07_RS02930 ← | NERD domain‑containing protein |
| 113,112 | G→C | D65E (GAC→GAG) | HKX07_RS00500 ← | immunity protein Tsi6 family protein |
| 2,735,454 | C→A | L81M (CTG→ATG) | tpsB1 → | two‑partner secretion system transporter TpsB1 |
| 2,740,526 | G→A | S1168N (AGC→AAC) | tpsA1 → | two‑partner secretion system exoprotein TpsA1 |
| 515,667 | A→C | W121G (TGG→GGG) | HKX07_RS02335 ← | DUF1232 domain‑containing protein |
| 515,672 | A→G | L119P (CTG→CCG) | HKX07_RS02335 ← | DUF1232 domain‑containing protein |
| 2,838,694 | G→A | A3349T (GCG→ACG) | HKX07_RS13190 → | non‑ribosomal peptide synthetase |
| 5,629,326 | T→G | E676D (GAA→GAC) | pilQ ← | type 4a pilus secretin PilQ |
| 5,629,474 | T→A | Y627F (TAC→TTC) | pilQ ← | type 4a pilus secretin PilQ |

**Supplementary Fig. S1:** Plaque morphology of phage SB on PAO1 (A) and PA4.6C (B). Uniform plaques with halos in 0.75% top layer LB agar.


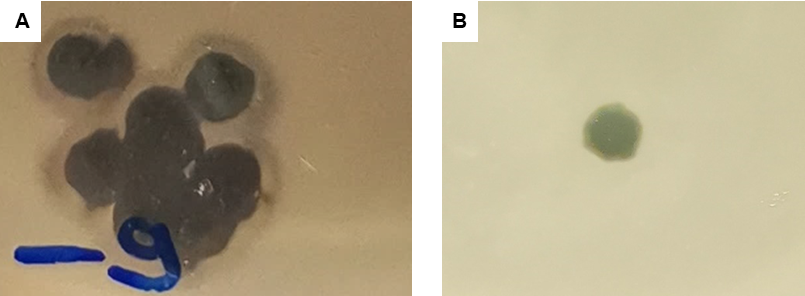


**Supplementary Fig. S2:** Phage stability under salt exposure. Stability of phage SB in 0.9% saline at 4 °C, under five different time durations (n=3 replicate measures per treatment). Estimates of PFU/mL obtained using the standard method of plaque assays.

**Supplementary Fig. S3:** Phage stability under serum and plasma exposure. Stability of phage SB in serum of one donor and plasma of one donor at 37 °C under three different time durations; n=1 replicate per treatment with error bars representing variation across 3 to 4 spot assay estimates. Serum or plasma was not tested for other phages potentially present in the respective donor.

**Supplementary Fig. S4:** Adsorption of phage SB to host cells. Adsorption of phage SB when mixed with host cells at initial MOI~0.01 and subjected to 37 °C incubated shaking. (**A**) Assays performed with n=4-6 replicates per 10-minute interval suggest adsorption occurs rapidly, within the first 10 minutes. (**B**) An additional assay performed with 3-minute intervals further suggests that adsorption occurs by ~6 minutes.


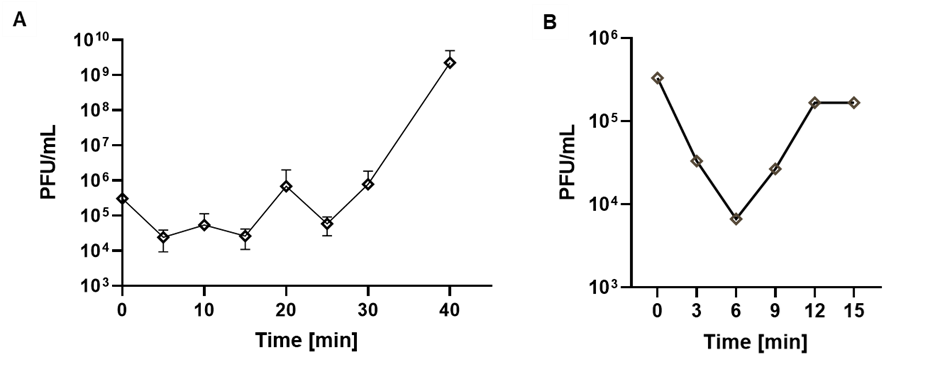


**Supplementary Fig. S5:** One step growth curve for phage SB population on host cells. Assays performed with 3 replicates at initial MOI~0.01, and with 37 °C shaking incubation.

**Supplementary Fig. S6:** Phage SB disrupts bacterial biofilm formation. Assays (n=6 replication) performed at initial MOI ~100 measured densities of PA4.6C host cells via (**A)** crystal violet staining, and (**B)** CFU counts.


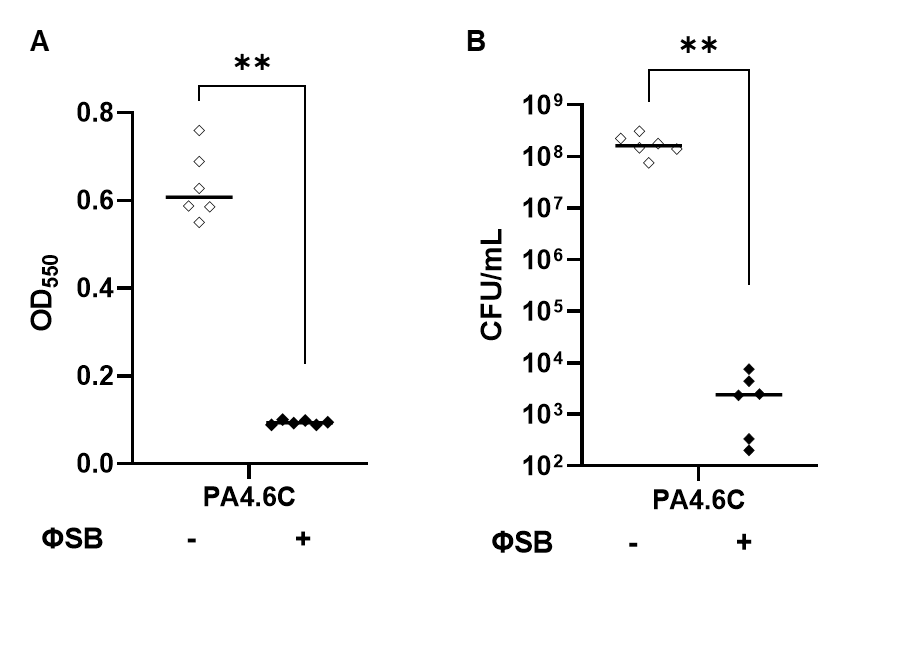


**Supplementary Fig. S7:** Phage SB does not alter bacterial biofilm formation of phage-resistant mutant PA4.6C-R. Assays (n=6 replication) performed at initial MOI~100 measured densities of PA4.6C-R cells via (**A)** crystal violet staining, and (**B)** CFU counts.


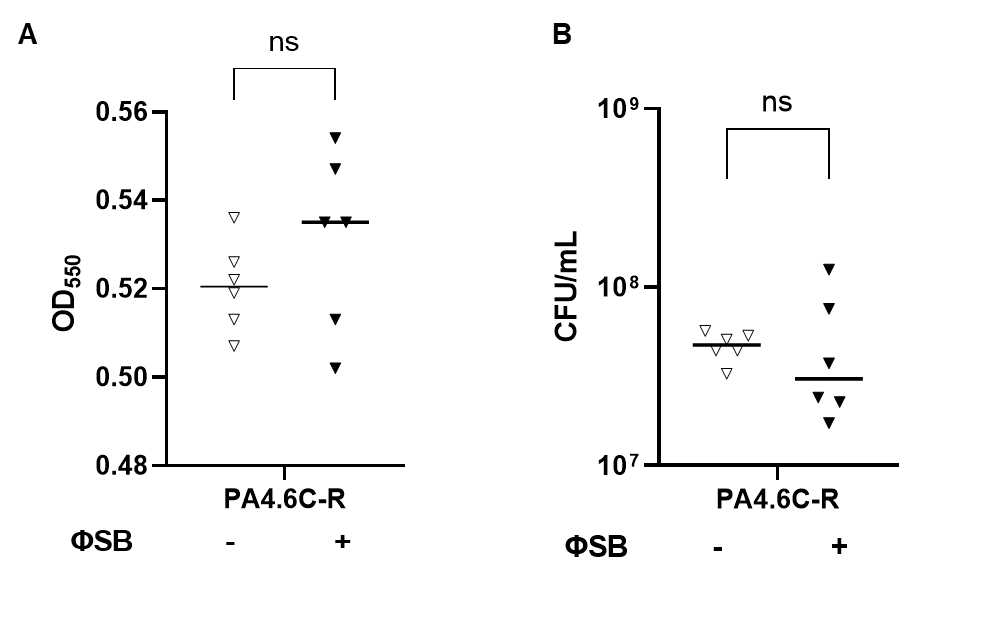


**Supplementary Fig. S8:** Comparison of biofilm formation capabilities of PA4.6C and PA4.6C-R bacteria. Assays (n=6 replication) performed at initial MOI~100 measured densities of cells via (**A)** crystal violet staining, and (**B)** CFU counts. Data presented in these graphs are also shown in Suppl. Figs. 5 and 6.


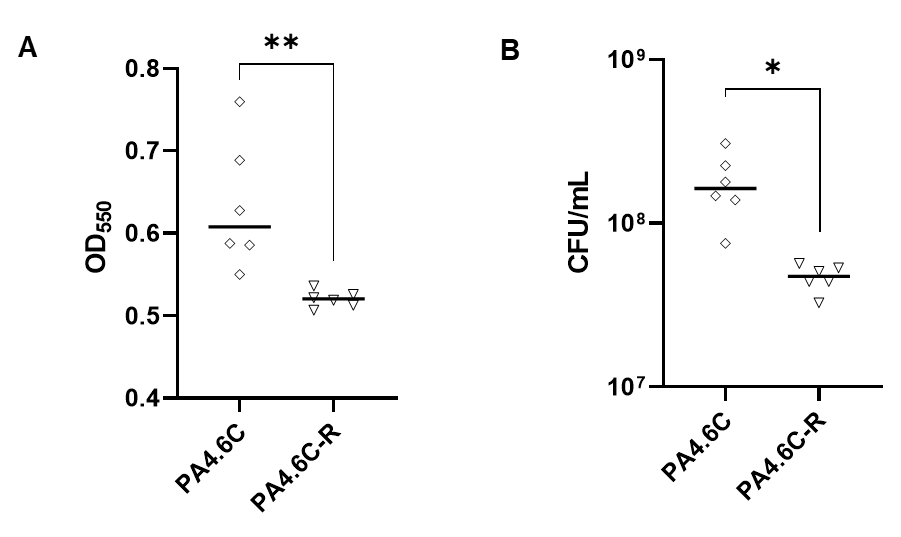


**Supplementary Fig. S9:** Comparison of antibiotic susceptibilities of PA4.6C and PA4.6C-R bacteria. (**A)** Minimum inhibitory concentration (MIC) tests (n=3 replicates) for each strain when exposed to five antibiotics. (**B)** Antibiotic susceptibility of each strain to Cefiderocol (n=1), when tested with 18 hours shaking at 37 °C. PA4.6C-R showed visible assemblages of cells in the suspension at 1.5µg/mL but no detectable growth when plated.


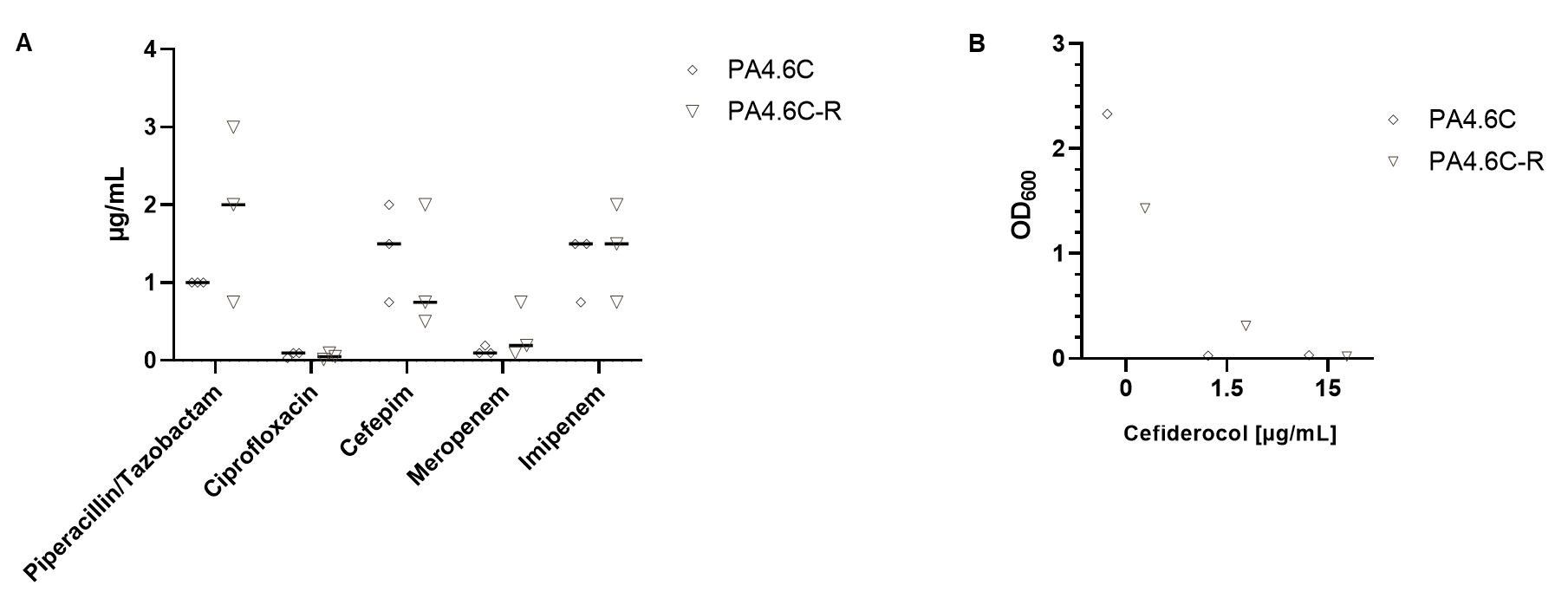


**Supplementary Fig. S10:** PA4.6C and PA4.6C-R strains do not differentially stimulate IL-8 production by THP-1 cells. Assays performed with n=3 replication. RPMI = cell culture medium, LB = bacterial medium, LPS = Lipopolysaccharide (*E. coli*, 100µg/mL).

**Supplementary Fig. S11:** Swimming and swarming motilities of PA4.6C and PA4.6C-R do not differ. **(A)** Swimming motility (diameter; n=4 replicates) estimated when bacteria were inoculated in the center of 0.25% agar plates. (**B)** Swarming motility (diameter; n=3 replicates) estimated when bacteria were inoculated on the surfaces of 0.75% agar plates.


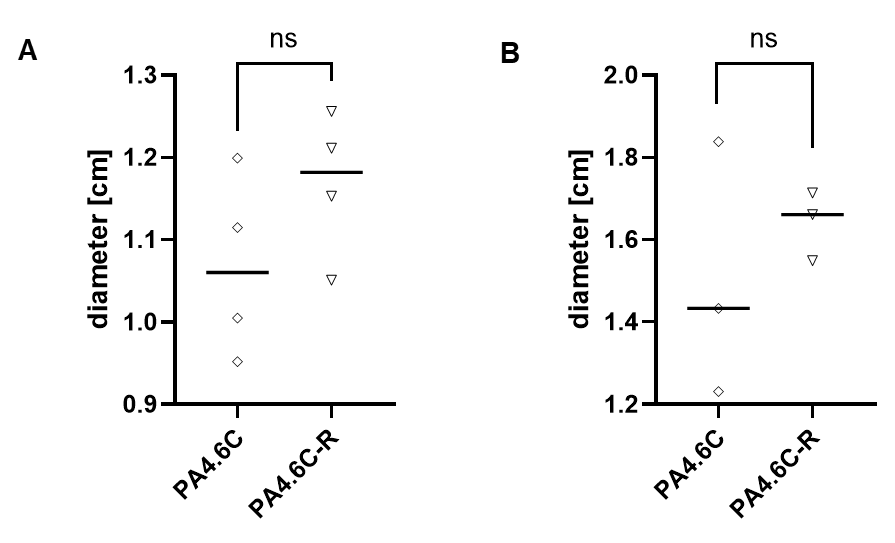

Supplement: Supplementary file 1 — Supplementary Information. [file 41598_2024_52192_MOESM1_ESM.docx]
